# Thyroid tumor-initiating cells: Increasing evidence and opportunities for anticancer therapy (Review)

**DOI:** 10.3892/or.2014.2978

**Published:** 2014-01-14

**Authors:** YONG-JU GAO, BO LI, XIN-YU WU, JING CUI, JIAN-KUI HAN

**Affiliations:** 1 Department of Nuclear Medicine, Qilu Hospital, Shandong University, Jinan, Shandong 250012, P.R. China; 2 Department of Nuclear Medicine, Henan Provincial People’s Hospital and the People’s Hospital of Zhengzhou University, Zhengzhou, Henan 450003, P.R. China

**Keywords:** cancer stem cells, tumor-initiating cells, cellular origin, anticancer therapy

## Abstract

Accumulating evidence supports the notion that thyroid cancer is initiated by tumor-initiating cells (TICs) (commonly known as cancer stem cells), which are thought to play a crucial role in malignant progression, therapeutic resistance and recurrence. Thyroid TICs have been isolated and identified using specific biomarkers (such as CD133), the side population, sphere formation and aldehyde dehydrogenase activity assays. Although their characteristics remain largely unknown, TICs provide an attractive cellular mechanism to explain therapeutic refractoriness. Efforts are currently being directed toward the identification of therapeutic strategies that could target these cells. The present review discusses the cellular origins of TICs and the main approaches used to isolate and identify thyroid TICs, with a focus on the remaining challenges and opportunities for anticancer therapy.

## 1. Introduction

Thyroid cancer is the most common endocrine malignancy, and its incidence continues to rise worldwide (1,2). Thyroid cancer is divided into four subtypes: 94% of cases are well-differentiated thyroid cancer that is derived from follicular epithelial cells and is categorized as either papillary thyroid carcinoma (PTC) or follicular thyroid carcinoma (FTC). The other 5% of cases are medullary thyroid cancer (MTC), a neuroendocrine tumor. The remaining 1% is anaplastic thyroid carcinoma (ATC), which is generally derived from de-differentiation of the differentiated thyroid cancer. Treatment of thyroid cancer is multifactorial, including surgery, radioiodine therapy and chemotherapy (1). Despite the widespread use of multimodal treatment, survival rates have not significantly improved in the past few decades (2). This suggests that some types of thyroid cancer are resistant to the current therapeutic options.

Recent progress in thyroid cancer biology has revealed that the histological appearance and biological behaviors of thyroid carcinoma often exhibit heterogeneity, with cells exhibiting distinct proliferative and differential capacities (3–5). Emerging evidence indicates that a rare subpopulation of cells purported to be cancer stem cells (CSCs) or tumor-initiating cells (TICs) drive thyroid cancer heterogeneity and contribute to the resistance to cancer therapy as observed in various cases (6–10). Moreover, clinical observations have revealed that TIC marker frequency in thyroid cancer is related to adverse outcomes (11). Thus, TICs have been indicated to play a crucial role in the malignant progression and therapeutic resistance of thyroid cancer. The present review summarizes and evaluates the recent evidence for the existence of TICs in thyroid cancer and the therapeutic strategies for targeting TICs based on the increasing biological knowledge of thyroid TICs.

## 2. Cellular origin and operational concept of thyroid tumor-initiating cells

**Figure 1:**
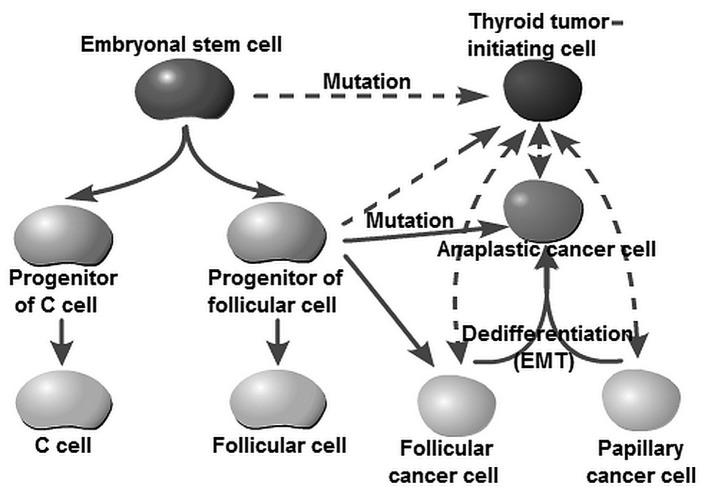
Cellular origin of thyroid tumor-initiating cells. Thyroid TICs may arise from normal stem cells, restricted progenitors, or more differentiated cells that have acquired stem-like characteristics through progressive genetic alterations and the phenomenon of EMT. TICs, tumor-initiating cells; EMT, epithelial-to-mesenchymal transition.

The cellular origins of thyroid cancer are still largely unknown. Yet, it has been speculated that different subtypes reflect cells of distinct origin at the time of tumor initiation. For decades, tumor initiation and development have been regarded as a multistep process, as reflected by the progressive genetic alterations that drive the transformation of normal fetal cells into highly malignant derivatives. The fetal cell carcinogenesis hypothesis postulated by Takano (12) and Takano and Amino (13) suggests that thyroid cancer cells are developed from remnant fetal thyroid cells. This model is partly similar to the current prevailing CSC hypothesis. The ‘CSC’ (often referred to as the TIC) theory is used to denote a cancer cell subpopulation with characteristics similar to those of normal stem cells (14,15). CSCs are characterized by their capacity to initiate tumor growth, self-renewal and differentiation by symmetric and asymmetric division (6,7,15). Zhu *et al* (16) and Barker *et al* (17) observed that CSCs were derived from normal stem cells if oncogenes were activated. It should be noted that the concept of a ‘CSC’ does not imply that the cell is derived from a normal stem cell. Chaffer *et al* (18) reported that basal-like epithelial cells could spontaneously de-differentiate into stem-like cells. Similar results showed that well-differentiated PTC or FTC cells could transform into undifferentiated ATC cells (5,19,20). Lan *et al* and Yasui *et al* as well as other research groups observed that more differentiated thyroid cancer cell populations acquire CSC properties through epithelial-to-mesenchymal transition (EMT) (21–24). These studies, therefore, raise the possibility that thyroid TICs may arise from restricted progenitors or more differentiated cells that have acquired self-renewing capacity. Thus, the originating tumor cells can be stem cells, progenitor cells or differentiated cells (Fig. 1). The American Association for Cancer Research (AACR) workshop stated that ‘cancer stem cells can only be defined experimentally by their ability to recapitulate the generation of a continuously growing tumor’ (25). Furthermore, the most widely accepted assay with which to validate a candidate CSC population is tumor initiation and serial transplantation in immunocompromised mice (26). Based on the consideration of cellular origin and functional identification, we herein recommend the use of the term ‘TIC’ instead of ‘CSC’ to describe this stem cell-like subpopulation within thyroid cancer.

## 3. Isolation and identification of thyroid tumor-initiating cells

Although the method is debated, putative TICs derived from thyroid carcinoma cell lines and tumor biopsy specimens were recently isolated using current prevailing cancer stem cell methods (27–30).

The approaches used to identify and isolate these putative TICs were based on the notion that TICs should have conserved stem and progenitor cell functions and phenotypes. TICs have been frequently isolated using specific markers from normal stem cells of the same organ (14,15). According to the literature, TICs can be isolated by the following four methodologies: flow cytometry-based cell sorting according to: i) TIC-specific cell surface markers; ii) aldehyde dehydrogenase activity; iii) ABCG2 efflux-pump-mediated Hoechst 33342 dye exclusion; and iiii) generation of spherical colonies in an ultra-low-attachment and serum-free culture. The following sections will discuss the various techniques that have to date been used in an attempt to isolate TICs in thyroid cancer as well as the limitations of these techniques.

### Surface markers

Surface markers have been used to identify and isolate adult stem/progenitor cells in human thyroid glands (31). Recent studies have been successful in using normal antigenic stem cell markers for the isolation of putative TICs in thyroid cancer. A promising marker that has been gaining popularity for the identification of thyroid TICs is CD133, also known as prominin-1 (32). CD133 is a 120-kDa cell surface protein comprising five transmembrane domains and was first described as a hematopoietic stem cell marker (33). Zito *et al* (34) found that CD133-positive cells comprised 64 and 57% of the cells within the undifferentiated ATC cell lines ARO and KAT-4, respectively. Sorted ARO CD133^+^ cells exhibit stem cell-like features such as increased proliferation, self-renewal ability, clone formation and resistance to chemotherapeutic agents (doxorubicin, cisplatin and etoposide). Friedman *et al* (35) detected CD133^+^ cells in two ATC cell lines, namely ARO (7.02%) and FRO (6.32%), but not in well-differentiated PTC cell lines (NPA and WRO). CD133^+^ cells harbor stem cell features characterized by high expression of stem cell gene Oct-4 and rapid long-term tumorigenesis. Ke *et al* (8) recently reported that CD133^+^ cells were present not only in the ATC cell line ARO (61.3%), but also in the PTC cell line CG3 (5%) and FTC cell line WRO (1.5%). A further study demonstrated that these CD133^+^ cells showed higher radioresistance and an undifferentiated status. Additionally, Zhu *et al* (36) suggested that the CD133^+^ subpopulation from MTC cell lines (MZ-CRC-1 and TT) exhibited the TIC features of self-renewal and multiple lineage differentiation.

Strikingly, the ATC cell line ARO used in the above-mentioned studies showed a significantly different CD133^+^ fraction ranging from 7.02 to 64% (8,34,35). In contrast, Friedman *et al* (35) did not detect CD133^+^ cells while Ke *et al* (8) found a 1.5% CD133-expressing fraction within the WRO PTC cell line. The frequency of CD133-positive cells appears to be highly variable among thyroid cancers of the same type. This issue may be related to the different cell culture environments and method standards in individual laboratories. In addition, a recent study provided evidence that the presence of TICs was frequently correlated with the tumor grade and clinical outcome (11). Thus, primary thyroid cancer cells should be used to verify the value of CD133 markers in the isolation of thyroid TICs. Furthermore, various surface markers such as Oct-4, GATA binding protein 4 (GATA4) and hepatocyte nuclear factor 4α (HNF4α) have been used to identify human thyroid stem/progenitor cells (31,37). Based on the notion that TICs frequently express specific markers from normal stem cells of a certain organ, it would be valuable to identify these markers in thyroid cancer.

### Side population assay

The side population (SP) assay is based on the differential potential of cells to efflux Hoechst dye (e.g., Hoechst 33342) via the ATP-binding cassette (ABC) family of transporter proteins expressed within the cell membrane (27). The ability of ABC transporters to rapidly efflux lipophilic fluorescent dyes *in vitro* serves as the basis of the SP assay and was originally identified in mouse bone marrow cells (38). SP cells were found, not only to have stem cell characteristics, but also to be enriched in a stem cell population (39). Hoshi *et al* (40) indicated that mouse thyroid SP cells are less differentiated and have characteristics of stem/progenitor cells. Thus, the SP assay has now emerged as a promising method for identifying stem-like cells in thyroid cancer. Mitsutake *et al* (41) reported that well-differentiated PTC (NPA) and FTC (WRO) cells or undifferentiated ATC (ARO and FRO) cell lines contained SP cells (excluding TPC-1 PTC cells) ranging from 0.02 to 0.25% of the total viable cell population. In another study by Zheng *et al* (9), ATC cell lines (SW1736, C643 and HTh74) were found to comprise 0.43 to 0.83% of SP cells, indicating that the SP fraction varies among thyroid cancer cell lines. The SP cells within thyroid cancer cell lines displayed TIC features characterized by overexpression of embryonic stem cell genes, high tumorigenic potential following transplantation into immunocompromised mice, increased self-renewal capacity (thyrospheres), generation of non-SP cells and resistance to chemotherapeutic agents (doxorubicin).

Theoretically, this method may be more suitable for enrichment of potential TICs because it is less restricted by tissue specificity than the cell surface makers discussed above. However, there are some notable limitations and concerns regarding the use of this technique to isolate putative TICs. First, Mitsutake *et al* (41) were not able to detect the SP fraction in the PTC cell line TPC-1, indicating that SP cells may represent only one of the putative thyroid TIC populations. Furthermore, Steuer *et al* (42) observed that Hoechst 33342 could induce the differentiation of mouse embryonal carcinoma cell lines (PCC3, P19 and PCC4); this effect may cause confounding errors when introduced to stem cell sorting. Adhikari *et al* (43) found that Hoechst 33342 arrests human glioma and squamous carcinoma cell growth and the cell cycle (late S and G2 phases), and increases cytogenetic damage (micronucleus formation) under ionizing radiation exposure. Therefore, Hoechst dye may limit the functional analysis of the enriched SP fraction.

### Sphere formation assay

The sphere-forming assay is widely used in stem cell biology as this is a relatively simple yet robust method for isolating and expanding stem cell populations (28). Since the original sphere formation assay was used to isolate neural stem cells, cells with stem-like characteristics have been isolated from human goiters and thyroid cancer cells by culturing them as non-adherent spheres and characterizing them as stem/progenitor cells. Pillai *et al* (44) and Malaguarnera *et al* (45) reported that thyrospheres isolated from PTC-derived cells have stem-like properties characterized by the expression of stem cell markers and low/absent thyroid-specific markers. Furthermore, Malaguarnera *et al* found that thyrospheres overexpress insulin receptor (IR) isoforms and the IGF receptor (IGF-IR) and that treatment with IGF increases the stemness features of thyrospheres. Li *et al* (46) observed that ATC cells from thyroid cancer patients also formed thyrospheres. These thyrospheres expressed stem cell markers and exhibited tumorigenic and metastatic features in an orthotopic mouse model.

However, there is little definitive information regarding which cancer cells are propagated under these non-adherent and serum-free conditions. Visvader and Lindeman (47)observed that the breast cancer cells from the sphere were no more tumorigenic *in vivo*, and that cells from malignant pleural effusions (from patients with breast cancer) did not give rise to tumors after more than 10 months following fat-pad implantation, despite generating spheres in culture. Therefore, the sphere-formation ability is not restricted to TICs and self-renewal cannot solely be defined in the context of a sphere assay.

### Aldehyde dehydrogenase activity

Aldehyde dehydrogenase (ALDH) is an NAD(P)^+^-dependent enzyme involved in the detoxification of intracellular aldehydes to weak carboxylic acids and is significantly more highly expressed in stem/progenitor cells (29,48,49). In several types of cancer, ALDH, which plays a crucial role in stem cell biology, has emerged as a valuable functional marker for the isolation of TICs (48–51). The basis of the ALDH assay is that intracellular ALDH converts the uncharged ALDH substrate BODIPY-aminoacetaldehyde (BAAA) into the negatively charged BAA^−^, which is retained intracellularly and causes the cell to become highly fluorescent. Thus, the ALDH-positive and ALDH-negative cells can be separated based on intracellular ALDH activity (29). Todaro *et al* (10) found that FTC, PTC and UTC cells derived from thyroid cancer tissue contain a small population of tumorigenic cells that can be prospectively identified through ALDH activity. Thyroid cells with high ALDH expression (ALDH^high^) possess the ability to self-renew and re-initiate serial transplantable tumors that recapitulate the phenotype and metastatic behavior of the parental tumors promoted by the activation of Met and Akt. In another study, Klonisch *et al* (52) identified a 17–38% ALDH1^+^ cell subpopulation with potential stem-like characteristics in the ATC cell line UTC-8505C. Additionally, Carina *et al* (53) recently observed that the ATC cell line SW 1736 contained a high percentage of ALDH-positive cells (10.4±2.1%) and exhibited high expression of several TIC markers (Sox-2, Oct4, Nanog, c-myc and SSEA4).

### Identification of thyroid tumor-initiating cells

**Figure 2:**
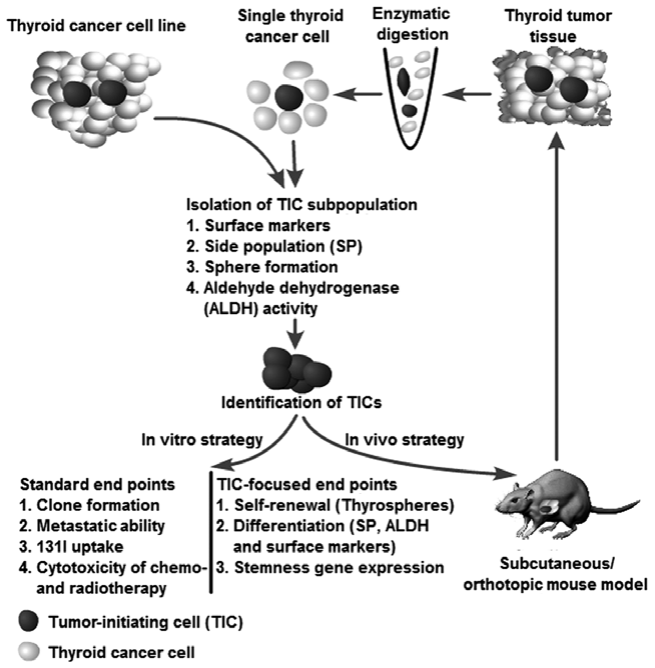
Identification of thyroid tumor-initiating cells. Thyroid TICs can be identified from patients with cancer or from certain cancer cell lines. Purified TIC functional identification includes *in vitro* and *in vivo* assays. For the *in vitro* assay, TICs can be studied according to standard endpoints (e.g., proliferation, metastasis and chemosensitivity and radiosensitivity) and TIC-focused endpoints (such as self-renewal, differentiation and stemness). For the *in vivo* strategy, tumor initiation and serial transplantation in a subcutaneous/orthotopic mouse model is the gold standard. TICs, tumor-initiating cells.

A series of strategies have been used to verify thyroid TICs isolated from patients with thyroid cancer and from cell lines using the methods mentioned above. The methods used to isolate TICs can also be used as strategies to identify putative TICs. For instance, the putative TICs isolated from an SP assay can validate their TIC characteristics through a sphere assay, ALDH activity, and the expression of surface markers (e.g., CD133) and vice versa. The strategies for identification of thyroid TICs are illustrated in Fig. 2.

Generally, putative TICs should demonstrate the ability to generate spheres in serum-free medium, even after serial passage, thus indicating that the cells have an extensive capacity for self-renewal. Furthermore, the cells should be able, not only to generate a heterogeneous tumor cell population, indicating multipotent potential, but also should have an unlimited capacity to reconstitute the primary tumor morphology, thus, demonstrating that heterogeneity is not simply a result of genetic instability. Moreover, surface markers can be used to identify their cellular origin, stemness and differentiation status. As an *in vivo* strategy, serial transplantation is the golden standard recommended by the AACR workshop to determine stem cell properties (25). The serial transplantation assay reflects the tumor-initiating ability of a certain cell population. If the putative TICs demonstrate the characteristics mentioned above, they can be recognized as TICs (Fig. 2).

It should be noted that not all TICs harbor these features. The different methods for the isolation of TICs may not overlap for the same tumor type or may do so to a limited degree. For instance, Todaro *et al* (10) observed that sphere-derived cells but not the ALDH^high^ thyroid cells from the same FTC5 tumors could initiate tumors in NOD/SCID mice. The most reasonable explanation for this observation may be related to the xenograft model itself. Species-specific differences and residual immune effects impair the tumor-initiating frequency. Furthermore, the ability of tumor initiation may be more accurately evaluated using an orthotopic transplantation to mimic the tumor environment as closely as possible. Finally, most studies of thyroid TICs have used thyroid cancer cell lines. Schweppe *et al* (54) reported that up to 42% of 40 types of thyroid cancer cells used in different laboratories were misidentified, redundant or cross-contaminated. In addition, repeated passaging of cell lines was found to lead to changes in characteristics and surface markers and the acquisition of genetic aberrations. Thus, observations made in thyroid cancer cell lines must be extended to primary tumors to validate their significance.

## 4. Therapeutic implications

**Figure 3:**
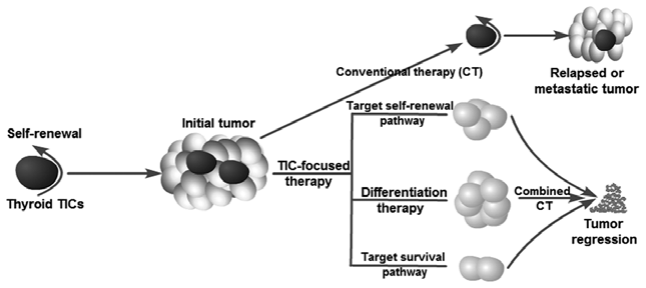
Therapeutic strategies by which to target tumor-initiating cells. Conventional therapy cannot destroy TICs. Thus, tumor relapse and metastasis are common. TIC-focused treatments include the targeting of self-renewal and survival signaling pathways as well as differentiation therapy. Tumor regression occurs when TICs are eradicated. TICs, tumor-initiating cells.

Current anticancer treatments are often able to destroy the bulk of a tumor but spare TICs. Thus, relapse or tumor recurrence is not rare after a primary therapeutic response or initial induction of tumor remission. The existence of TICs explains the phenomenon observed in clinical therapy. Zheng *et al* (9) and Ke *et al* (8) recently found that the failure of chemotherapy (doxorubicin) or radiotherapy (10 Gy) to eradicate thyroid cancer cells may be due to the ineffective targeting of thyroid TICs. An ideal therapeutic strategy should not only kill differentiated cancer cells (as conventional therapy does) but should also specifically destroy thyroid TICs simultaneously. Thus, the focus on thyroid TICs has significant clinical implications for the development of novel therapeutical strategies for refractory thyroid cancer (such as lack of sufficient radioiodine uptake and resistance to current chemotherapy) by targeting TICs. In fact, several studies have investigated possible strategies by which to target thyroid TICs and overcome resistance to current therapeutic options (Fig. 3). Potential approaches to killing TICs include blocking self-renewal signaling pathways, inducing tumor cell differentiation and inhibiting survival mechanisms, thus overcoming chemoresistance and radioresistance.

### Targeting self-renewal signaling pathways

Approaches to blocking the self-renewal of TICs represent rational therapeutic strategies for cancer prevention and treatment. The signal transducer and activator of transcription 3 (STAT3) pathway has been shown to be required for self-renewal of TICs in several types of cancers, including hepatoma and glioblastoma (55,56). Microarray bioinformatic analysis from ATC-derived cells recently revealed that the STAT3 pathway plays an essential role for maintaining the self-renewal of ATC-CD133^+^ cells, since blockage of STAT3 activity using cucurbitacin I was found to significantly diminish TIC features such as stemness gene expression, and induce their differentiation. Cucurbitacin I plus radiochemotherapy suppressed CD133^+^ cell survival and tumorigenesis *in vitro* and *in vivo*, respectively (57). Sox-2 is a well-established regulator in the maintenance of adult tissue homeostasis and regeneration (58). Accumulating data indicate that Sox-2 expression is abnormally elevated in thyroid TICs, thus, serving as a therapeutic target. Carina *et al* (53) found that the silencing of Sox-2 decreased the expression level of key stemness genes (Nanog and Oct4) and increased the sensitivity to chemotherapeutic agent (cisplatin and doxorubicin)-induced cell death in the ATC SW1736 cell line. In addition, Malaguarnera *et al* (45) reported that overexpression of the insulin receptor and IGF-I receptor in thyrospheres may sustain their self-renewal capacity and promote TIC survival. Metformin suppression of the self-renewal of derived thyroid TICs appears to be related to inhibition of the insulin/IGF and AMPK signaling pathways, but this finding still requires further investigation (59). The thyrotropin receptor, which has been found to be highly expressed in CD133^+^ cells, also plays a crucial role in maintaining stemness, thus serving as another potential target (35). Finally, Zhu *et al* (36) found that the RET tyrosine kinase receptor gene (RET) signaling pathway, which plays a fundamental role in stem cell maintenance and development of MTC contributes to tumor progression through facilitation of TIC (thyrosphere) renewal. Knockdown of RET expression reduced the sphere formation ability (a cardinal features of TICs) of MTC cells. This suggests that targeting of the RET receptor offer another opportunity to eradicate TIC populations (60,61).

### Differentiation therapy

TICs facilitate tumor growth through enhanced self-renewal and limited differentiation capacity. Therefore, an alternative approach to the eradication of TICs may be achievable by overcoming the blockage of differentiation by TICs. In the 1970s, this approach led to the identification and clinical use of all*-trans*-retinoic acid for patients with acute promyelocytic leukemia (62) and metastatic thyroid cancer (63). Tseng *et al* (57) found that cucurbitacin I suppressed the proliferation and TIC properties of CD133^+^ cells through induction of their differentiation into CD133^−^ cells characterized by upregulation of thyroid-specific genes (NIS, TPO and Tg) and enhanced radioiodine uptake. The effects of cucurbitacin I on normal thyroid stem/progenitor cells remain unclear. Of note, differentiation therapy should involve selective targeting of TICs while having no effect on normal stem cells.

### Targeting of other implicated molecular pathways

The cell surface molecule CD44 may also be an important target. CD44 is a transmembrane glycoprotein involved in tumor cell proliferation and metastasis (64). De Falco *et al* (65) revealed that the CD44-CERB signaling pathway is required to sustain thyroid cancer cells, as evidenced by the finding that knockdown of CD44 obstructed cell proliferation. CD44 was recently recognized as a promising marker of TICs in many cancer types, including thyroid cancer (10,66). Thus, CD44 serves as a potential cell surface marker for targeting TICs. Other described targets include ABCG2 (breast cancer-resistance protein, BRCP1) and ABCB1 (P-glycoprotein, MDR1), which were found to be overexpressed in doxorubicin-resistant ATC cancer cells (9). ABCG2 and MDR1 are the best-characterized drug transporter proteins belonging to the ABC family that function to establish the SP phenotype (27,67,68). TICs express high levels of ABC drug pumps, which enable the efflux of drugs from cells, thus serving to protect them from chemotherapeutic agents (68,69). Silencing of ABCG2 expression increases ATC cell sensitivity to cisplatin and doxorubicin treatment (53). Finally, Todaro *et al* (10) reported that enhanced cell survival and migratory ability are associated with increased Met and Akt expression in ALDH^high^ thyroid cancer cells, whereas silencing of these genes was found to decrease the tumorigenic and metastatic capacities.

### ^131^I therapy and thyroid tumor-initiating cells

^131^I therapy is regularly used following surgery as a part of thyroid cancer management. Feng *et al* (70) observed that undifferentiated thyroid cancer cell populations were enriched after ^131^I treatment, which indicates that TICs involved in thyroid cancer may be resistant to ^131^I therapy. Failure to accumulate iodide and resistance to ionizing radiation (IR) may be the two main reasons for TIC insensitivity to ^131^I therapy. Undifferentiated thyroid carcinomas have an absent or decreased ability for iodide uptake due to decreased or undetected sodium-iodide symporter (NIS) expression (71). As mentioned in the section, ‘Differentiation therapy’, cucurbitacin I induces CD133^+^ cell differentiation by upregulation of NIS expression and thus enhances radioiodine uptake (57). Therefore, differentiation therapy may be a promising method for restoration of the ^131^I uptake by TICs. A phase II study used retinoids as redifferentiation agents to increase the iodine uptake for metastatic thyroid cancer (63). Furthermore, accumulating evidence suggests that TIC resistance to ionizing radiation results from quiescence propensity, enhanced DNA repair, upregulated cell cycle control mechanisms, mechanisms of free-radical scavenging, and specific interaction with the stromal microenvironment (72,73). Tseng *et al* (57) and Ke *et al* (8) reported that CD133^+^ thyroid cancer cells exhibit reduced IR-induced cell death, particularly apoptosis. The critical mechanisms determining the resistance of thyroid TICs to radiation remain elusive and warrant further investigation. TICs can be effectively eradicated by combined methods that overcome IR resistance and restore radioiodine uptake with ^131^I therapy.

## 5. Future direction

Although accumulating evidence supports the existence of TICs in thyroid cancer, and potential therapeutic strategies for targeting thyroid TICs are currently underway, some unanswered questions remain. First, the most basic cellular origins of thyroid TICs are transplantations from other malignancies. Thus, the origin of thyroid TICs should be explored in further studies to prevent tumor initiation. Second, the TIC fractions in thyroid cancer are highly impure, and the reported frequencies in the same tumor types have varied enormously among different research groups. This reflects the fact that TICs themselves are heterogeneous. This highlights the importance to identify additional specific markers or to use combinations of markers. Finally, the existence of TICs may depend on the specific niche. Accumulating evidence suggests that the tumor microenvironment is necessary for sustaining the stem-like properties of TICs (74,75). Thus, it is desirable to establish more definitive methods by which the tumor microenvironment can be mimicked when isolating and identifying TICs. On the other hand, the TIC niche could, therefore, be a therapeutic target for the design of therapies aimed at eradicating thyroid TICs. Studies concerning the thyroid TIC niche are rare, and thus this subject should be investigated in-depth. An increased understanding of thyroid TIC biology will provide an important framework for drug discovery and cancer treatment with the potential to identify novel antitumor activities, impact cancers with undifferentiated phenotypes, and yield long-term benefits for patients with thyroid cancer.
